# Clinical Presentation and Characteristics of the Upper Extremity in Patients with Musculocontractural Ehlers–Danlos Syndrome

**DOI:** 10.3390/genes13111978

**Published:** 2022-10-29

**Authors:** Fumihiro Isobe, Masanori Hayashi, Rena Kobayashi, Manami Nakamura, Tomoki Kosho, Jun Takahashi

**Affiliations:** 1 Department of Orthopaedic Surgery, Shinshu University School of Medicine, Matsumoto 390-8621, Japan; 2 Shinshu University School of Medicine, Matsumoto 390-8621, Japan; 3 Department of Medical Genetics, Shinshu University School of Medicine, Matsumoto 390-8621, Japan; 4 Center for Medical Genetics, Shinshu University Hospital, Matsumoto 390-8621, Japan; 5 Division of Clinical Sequencing, Shinshu University School of Medicine, Matsumoto 390-8621, Japan; 6 Research Center for Supports to Advanced Science, Shinshu University, Matsumoto 390-8621, Japan

**Keywords:** musculocontractural Ehlers–Danlos syndrome, elbow osteoarthritis, radial head dislocation

## Abstract

Musculocontractural Ehlers–Danlos syndrome (mcEDS) is a subtype of EDS caused by defective dermatan sulfate biosynthesis, characterized by multiple malformations (craniofacial features, ocular and visceral malformations) and progressive cutaneous, skeletal, vascular, and visceral fragility-related manifestations. Repeated dislocations and deformities of the joints due to joint relaxation are observed, causing serious damage to the musculoskeletal system of the whole body; however, the motor function of the upper limbs and the morphology of the bone joints have not been systematically investigated. In this study, we present a detailed and comprehensive report on upper limb lesions of 13 patients with a mean age at the first visit of 21 years. Twelve patients (92.3%) had a history of dislocation. Eleven patients (84.6%) had shoulder dislocations, and two patients (15.4%) had elbow dislocations. Four patients (30.8%) had elbow osteoarthritis, and three patients (23.1%) had distal radioulnar joint (DRUJ) osteoarthritis. The phalanges and metacarpals are thin, and the ratio of medullary cavity of the metacarpal bone decreases with age. As bone and joint deformity progresses, patients with mcEDS should be recommended to receive regular follow-up, including radiology. The present findings suggest an important role for dermatan sulfate in the maintenance of the skeletal system.

## 1. Introduction

Ehlers–Danlos syndrome is a group of diseases that develop due to congenital abnormalities in connective tissue collagen and collagen-modifying enzymes, and the diseases are characterized by hyperextensibility of skin and hypermobility of joints, in addition to the fragility of various tissues. Thirteen subtypes are commonly classified based on clinical characteristics, mode of inheritance, and molecular evaluation [1]. One of the EDS subtypes is musculocontractural Ehlers–Danlos syndrome (mcEDS). The subtype is further classified into mcEDS-*CHST14*, which is caused by dermatan 4-*O*-sulfotransferase 1 deficiency based on *CHST14* gene mutation, and mcEDS-*DSE*, which is caused by dermatan sulfate epimerase deficiency. Both subtypes result in the systemic depletion of dermatan sulfate [2,3,4,5]. Clinical features of mcEDS-*CHST14* include distinct craniofacial features, multiple joint contractures, progressive connective tissue weakness (i.e., skin laxity and giant subcutaneous hematoma), repetitive joint dislocations and deformities due to joint laxity, and severe damage to the locomotor system of the whole body [3]. Although the clinical presentation of mcEDS-*DSE* is similar to that of mcEDS-*CHST14*, joint-related symptoms (dislocation and hypermobility), cutaneous features (hyperextensibility and frailty), hypotonia, and gross motor developmental delays are less common than in mcEDS-*CHST14* [5].

The key characteristics of human upper extremity motor function are the performance of complex tasks using hands in addition to the mobility of the shoulder and elbow joints that facilitates the extension of arms from the head, neck, face, and lower extremities that allow a wider range of movement. However, there have been no detailed reports on upper extremity motor function and bone joint morphology in patients with mcEDS. In this study, we aimed to clarify the clinical characteristics and motor function of the upper extremities in patients with mcEDS based on upper extremity examination findings distal to the elbow joint, plain radiographic findings, and patient-reported functional evaluation scores.

## 2. Materials and Methods

### 2.1. Patients

We recruited 13 patients from 11 families who had biallelic mutations in *CHST14* or *DSE*. These patients consisted of 12 patients with mcEDS-*CHST14* and 1 patient with mcEDS-*DSE*. The cohort included six male and seven female patients with a mean age of 20.8 ± 12.4 years (range 4–45 years) at the first visit to our hospital (Table 1).

### 2.2. Protocol

Medical examinations were performed by experienced orthopedic surgeons (F.I., M.H.) to examine the presence of motion pain or tenderness on each shoulder, elbow, wrist, and hand of the patients. The range of flexion, extension, supination, and pronation was also recorded with a goniometer. Varus–valgus stabilities of the elbow were evaluated. The grip strength and side pinch strength were measured once on both hands using a Jamar Hydraulic Hand Dynamometer and Jamar Pinch Gauge (Sammons Preston, Rolyon, Bolingbrook, IL, USA) and were defined as the value obtained by dividing the sum of the left and right measurements by 2, respectively [6]. The Disability of Arm, Shoulder, and Hand (DASH) questionnaire is widely used as a subjective method of evaluation for upper extremity disorders [7]. QuickDASH is an abbreviated 11-item version of the 30-item DASH, whereby higher scores out of 100 are indicative of more severe disability [8]. Japanese-language versions of the QuickDASH questionnaire were collected at the day of examination (Table 2) [9].

### 2.3. Radiological Measurement

Anteroposterior and lateral radiographs of the elbow and posteroanterior and lateral radiographs of the wrist and hand were routinely obtained. 

#### 2.3.1. Elbow

**Figure 1:**
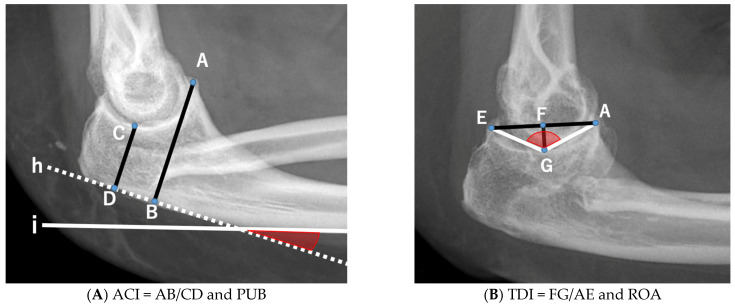
Index measurement of the elbow joint. A: tip of the coronoid process, AB: linear distance between point A and the posterior olecranon line, CD: minimal proximal ulna trochlear height, E: tip of the olecranon process, F: midpoint of the segment from AE, G: deepest point of the trochlear notch (determined by the intersection of a line perpendicular to AE and passing through point G and the trochlear notch), h: posterior olecranon cortex line, i: posterior ulna cortex line. (**A**) Proximal Ulnar Bow (PUB) is determined by lines h and i. (**B**) Radiographic Opening Angle (ROA) is determined by lines AG and GE. ACI, Anterior Coverage Index; TDI, Trochlear Depth Index.

Two primary indices and two derived angles comprising the Anterior Coverage Index (ACI), Trochlear Depth Index (TDI), Proximal Ulnar Bow (PUB), and Radiographic Opening Angle (ROA) were measured on lateral elbow radiographs (Figure 1) [10,11].

Linear measurements were obtained from a 7-point radiographic coordinate system. The coronoid height (distance from point A to B), minimal trochlear height (C to D), and segments spanning from A to E and F to G were measured. These measurements were used to calculate the following two primary indices:

1. Anterior Coverage Index (ACI): defined as the ratio between the coronoid height (point A to B) and the minimal proximal ulna trochlear height (C to D).
2. Trochlear Depth Index (TDI): defined as the ratio between the proximal ulna trochlear notch depth (F to G) and olecranon–coronoid distance (A to E).

Furthermore, two derived angles were measured:

1. Proximal Ulnar Bow (PUB): defined as the angle between the ulnar diaphysis and olecranon.
2. Radiographic Opening Angle (ROA): defined as the angle between lines from the deepest point of the trochlear notch to coronoid (A to G) and olecranon (G to E).

#### 2.3.2. Wrist

**Figure 2:**
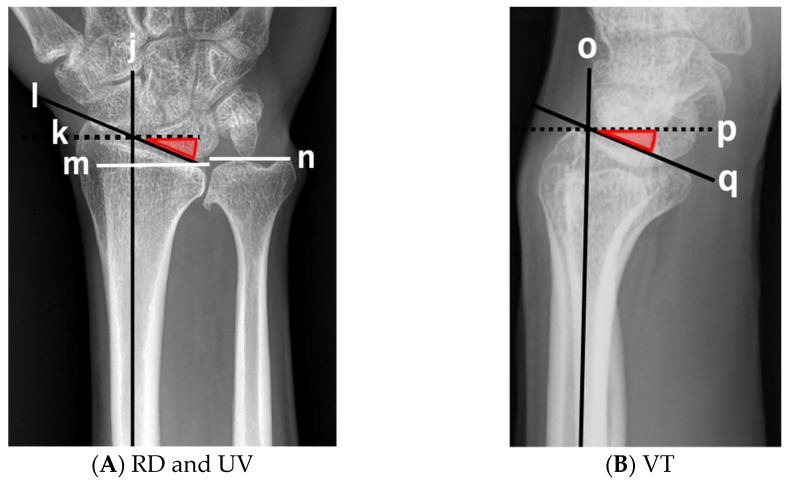
Measurement in the wrist joint. j: long axis of the radius in posteroanterior view, k: line perpendicular to long axis of radius in posteroanterior view, l: tilt of the distal end of the radius on posteroanterior radiograph, m: distal end of the radius, n: distal end of the ulna, o: long axis of the radius in lateral view, p: line perpendicular to long axis of the radius in lateral view, q: tilt of the distal end of the radius on lateral radiograph. (**A**) Radial deviation (RD) is determined by lines k and l. Ulnar Variance (UV) is the sum of lines m and n. (**B**) Volar tilt (VT) is determined by lines p and q.

Radial deviation (RD) and ulnar variance (UV) were measured in the posteroanterior view of the wrist. Volar tilt (VT) was measured on the lateral view of the wrist (Figure 2) [12]. RD and VT of the distal radial articular surface were measured in degrees. UV was defined as the distance between the distal ends of the radius and ulna.

#### 2.3.3. Hand

**Figure 3:**
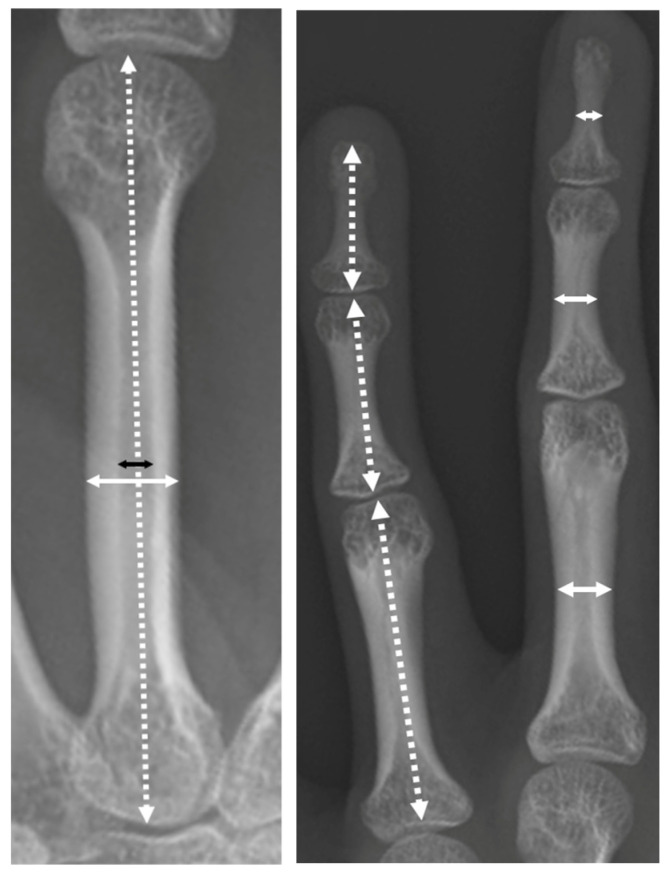
The two-headed arrows of the white dotted line indicate the length of the metacarpal bones and distal, middle, and proximal phalanges. The two-headed arrows of the white solid line indicate the width of the metacarpal bones and distal, middle, and proximal phalanges. The black two-headed arrows indicate the width of the third metacarpal medullary cavity.

The length and width of each phalanx and metacarpal bone and the width of the third metacarpal medullary cavity were measured on posteroanterior radiographs of the hand, and the ratio of the medullary cavity of the metacarpal bone was measured (width of medullary cavity/metacarpal) and calculated (Figure 3).

Finally, the presence of dislocation, deformity, and osteoarthritis (OA) was diagnosed by an orthopedic surgeon from coronal and sagittal radiographs of the elbow and wrist joint, including the scaphotrapeziotrapezoidal joint, radiocarpal joint, distal radioulnar joint, and hand. OA was defined as Kellgren–Lawrence grade ≥ 2 of the elbows, wrists, and hands [13]. Radiographic measurements were taken twice by the same examiner with an interval of 1 week or more, and the mean value was calculated.

## 3. Results

### 3.1. Medical Examination and Consultation 

One patient had a history of fracture, and twelve patients (92.3%) had a history of dislocations. Eleven patients (84.6%) had shoulder dislocations, two patients (15.4%) had elbow dislocations, and seven patients had patellar dislocations (Table 1). Ten patients (76.9%) had a history of two or more dislocations. One mcEDS-*DSE* patient had no history of dislocation.

None of the patients exhibited pain. Motion pain was observed in three patients (two elbows, one hand), and tenderness was observed in two patients (two elbows). There was no varus–valgus instability of the elbow joint. One patient had difficulty lifting the shoulder. Eight patients (61.5%) had extensor tendon dislocation at the metacarpophalangeal joint, and four patients (30.8%) had swan neck deformity. 

Patients under the age of 18 had a mean range of motion of the elbow joint of 145° in flexion and 11° in extension, with a tendency toward hyperextension. Adult patients (18 years and older) had a mean range of motion of the elbow joint of 143° in flexion and −4° in extension, with a limited range of motion in extension. In addition, the mean range of motion of the wrist was 87° in flexion and 71° in extension, which was normal regardless of age. The mean range of motion of the thumb was 34° in interphalangeal (IP) joint flexion and −2° in IP extension, showing a significant limitation in flexion. Furthermore, the mean range of motion of the index finger, middle finger, ring finger, and little finger were 34°, 41°, 42°, and 44° in distal interphalangeal (DIP) joint flexion, and −16°, −23°, −19°, and −18° in DIP extension, indicating a significantly limited range of motion. Adult patients had a mean grip strength of 8.2 kg and side pinch strength of 3.3 kg. One mcEDS-*DSE* patient had no severe loss of grip and side pinch strength.

### 3.2. Radiological Examination 

The ACI, TDI, PUB, and ROA measured at the elbow joint, and RD, VT, and UV measured at the wrist are summarized in Table 3. TDI and ROA were not measured in patients younger than 12 years, since the epiphyseal nucleus of the olecranon has not yet appeared. Due to the difficulty and pre-closure of the epiphyseal line of the radius before age 18 years, wrist parameter measurements were recorded as reference values. ACI (mean ± SD) was 1.70 ± 0.26, TDI was 0.39 ± 0.07, PUB was 11.5 ± 7.5, and ROA was 103 ± 11. RD was 24.6 ± 3.8, VT was 18.2 ± 6.0 and UV was 1.2 ± 2.6.

**Figure 4:**
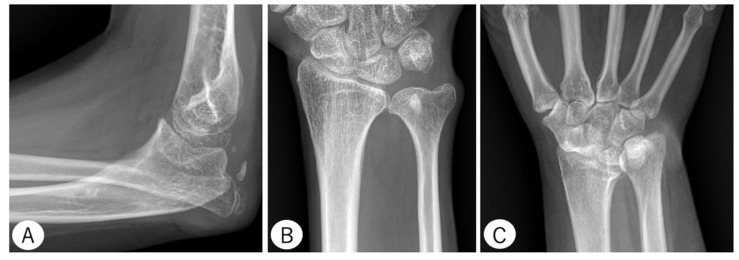
Radiographs of example cases. Patient 3 at age 11 years shows an angularly deformed proximal ulna and posteriorly dislocated radial head (**A**). Patient 12 at age 39 years shows a narrow ulnar diaphysis and an enlarged distal area (**B**). Patient 11 at age 31 years shows a fused scaphoid and trapezium (**C**).

Six patients (46.2%) sustained a radial head dislocation (four adults, two children). In addition, 5 patients (38.5%) exhibited ulnar head deformity (five adults), and two patients (15.4%) had unilateral fusion of the scaphoid and the trapezium (two adults) (Figure 4). Four patients (30.8%) had elbow OA, and three patients (23.1%) had distal radioulnar joint (DRUJ) OA. OA was only observed in adult patients, and hand OA was not observed.

The phalanges and metacarpals are thin, and the ratio of medullary cavity of the metacarpal bone decreases with age (Table 4 and Table 5).

### 3.3. QuickDASH Patient-Reported Outcome Measure for Assessing the Upper Extremity

The QuickDASH scores of all patients are shown in Table 6. The mean score was 34.5 (4.5–54.5). Children were unable to perform Component 2 (household chores) and Component 4 (wash your back); however, adults were able to perform these tasks to some extent. Pain domains of Components 9–11 tended to increase in adults. Patient 12 with mcEDS-*DSE* had a score of 15.9, which was better than the mean score.

## 4. Discussion

In this study, we investigated the clinical characteristics and motor function of the upper extremity distal to the elbow joint in patients with mcEDS. Ten patients (76.9%) had a history of two or more dislocations. Radiographic findings showed morphological abnormalities of the bone in most patients, and 70% of patients exhibited a shallow trochlear notch. The ratio of medullary cavity to metacarpal width decreased with age. Upper extremity function scores were shown to decrease in most patients. One mcEDS-*DSE* patient had no history of dislocation, no severe loss of grip strength, and a QuickDASH score of 15.9, which was better than the mean score.

Eight patients (66.7%) sustained extensor tendon dislocations at the metacarpophalangeal joint on examination. The extensor tendon of the MP joint is a structure in which the extensor tendon of the finger, central slip, and sagittal band on the radial and ulnar sides support a construct that is akin to a balancing toy; therefore, if one of the sagittal bands is relaxed, the central slip can dislocate in the opposite direction, resulting in an extensor tendon dislocation [14]. Relaxation of the sagittal band is also common in mcEDS. Four patients (30.8%) had swan neck deformity, which may be due to congenital laxity of the volar plate of the PIP joint. Patients under the age of 18 tended to hyperextend the elbow joint; however, adult patients developed OA and limited range of motion in elbow extension. The IP joints of all fingers were in a flexed position, and the limitations in range of motion are an important clinical feature of this disease. Although the mean grip strength of healthy Japanese adults has been reported to be 42.0 kg for males and 25.5 kg for females [15], the grip strength of patients with this disease was clearly reduced compared with the healthy adult population. 

Joint subluxation and dislocation are common in those with Ehlers–Danlos syndrome, because joint laxity generally increases the range of motion of joints. The frequency of dislocation in patients with mcEDS-*CHST14* who participated in this study was highest in the order of shoulder joint, patella, and elbow joint. Among them, the physical features that caused the dislocation of the elbow joint can be inferred from the physical findings and examination results. Elbow joint dislocation is classified based on the direction of the proximal radioulnar joint position in relation to the distal humerus, including anterior, posterior, posteromedial, posterolateral, lateral, and divergent dislocations. Posterior and posterolateral dislocations account for 90% of all dislocations [16]. King et al. reported that the dislocation of the elbow joint is caused by (1) laxity or injury of the collateral ligament, (2) laxity or rupture of the joint capsule, and (3) shallow trochlear notch [17]. Shallow trochlear notch includes congenital hypoplasia of the trochlear notch and those secondary to trauma-induced coronoid process fractures. Considering that there was no clear varus–valgus instability of the elbow joint and neither of the two cases with a history of elbow dislocation had a history of trauma such as fractures, we believe that there was no laxity of the collateral ligament. Therefore, congenital laxity of the joint capsule or shallow trochlear notch may have potentially caused the dislocation. Bony congruence in the ulno-humeral joint contributes to elbow stability. A shallow trochlear notch may be caused by coronoid process fractures; however, since coronoid process fractures may be overlooked, parameters are measured on plain radiographs of the elbow joint to reduce these oversights. In a study using fresh-frozen cadaver specimens of upper limbs from human donors, Luceri et al. found that the ACI decreased from 1.90 prior to a fracture to 1.58 following a fracture of the coronoid process, and the TDI decreased from 0.45 prior to a fracture to 0.39 following a fracture [10]. A study by Kilgus et al. reported that the elbow instability group showed a significantly larger opening angle (94.1° vs. 88.5°) [11]. Compared with healthy subjects described in a study by Luceri et al., the mean ACI and TDI measured in this study were lower than that of healthy subjects, and the ROA was higher than those of healthy subjects, suggesting that many cases are consistent with patients who have a shallow trochlear notch. 

The coronoid process plays an important role in maintaining the anterior stability of the elbow joint. The loss of coronoid process height leads to elbow joint instability [18]. Elbow joints with decreased anterior stability can lead to a posterior translation of the ulna that can induce a posterior dislocation of the elbow. Seki et al. conducted a biomechanical study and reported that resistance to varus and posterolateral rotational forces is provided by the tension of the lateral collateral ligament complex and the joint reaction force of the coronoid process [19]. Moreover, Hull et al. reported that the resistance to varus decreased when more than 50% of the coronoid process was lost, concluding that the coronoid process is an important varus stabilizer [20]. In this study, two patients with mcEDS-*CHST14* and a history of elbow joint dislocation were both consistent with patients who have a shallow trochlear notch. Based on the above, mcEDS-*CHST14* patients with a shallow trochlear notch and no history of dislocation are likely to develop posterior or posterolateral dislocation of the elbow joint in the future. These types of dislocations generally occur when a person falls onto an outstretched hand; therefore, educational activities and preventative measures may help reduce the frequency of dislocation by urging patients to pay attention to the mechanism of the injury. In addition, the use of braces that limit elbow extension may be effective in reducing the incidence of elbow joint dislocation in patients with elbow hyperextension.

Six patients, accounting for approximately half of the patients, sustained a dislocation of the radial head. Of these dislocations, all were posterior dislocations. The PUB, a measure of proximal ulnar kyphosis deformity, tended to be large in cases of radial head dislocation. Osteoarthritis of the elbow develops in adults due to a history of these alignment abnormalities and dislocations [21]. In this study, elbow OA occurred in four out of six patients aged 20 to 50 years (66.7%), which is an extremely high incidence rate even when considering the 25% prevalence of elbow OA in the general population aged from 50 to 90 years [22]. Three adult patients had DRUJ OA. All radiographic parameters of the wrist in five teenage patients were within normal limits, but UV plus was shown to be prominent in adults. Due to the dislocation of the radial head, the ulna becomes relatively long and may lead to DRUJ OA.

Although fusion of the scaphoid and trapezium were observed in two patients, this is a rare congenital defect with a prevalence of less than 0.1% [23]. The carpal bones are formed during embryogenesis by separation and cavitation of the cartilaginous precursors of the carpals. When there is an incomplete separation or cavitation, a carpal coalition is formed [24]. Considering the prevalence in the general population, we believe that the coalition of the carpal bones is a common complication in those with mcEDS.

In this study, we investigated the mean length and width of phalanges and metacarpals of adult mcEDS patients by gender. The length of phalanges and metacarpals of adults in this study was similar to that of the general population in Japan [25]; however, the width of the bone tended to be narrower in this study. In addition, the ratio of the medullary cavity to the metacarpal width decreased with age. The ratio tended to decrease sharply in the late teens, while leveling off or decreasing slightly after adulthood. Similar to a report by Kosho et al., this study found that narrowing of the medullary cavity of the metacarpal bone progresses with bone growth, and that dermatan sulfate may be associated with bone formation [26,27].

The mean QuickDASH score was 34.5, revealing that the majority of mcEDS-*CHST14* patients demonstrated reduced upper extremity function. On the other hand, all patients with elbow and DRUJ OA had pain domains of two or less, indicating that patients with OA did not necessarily suffer from severe elbow and wrist pain. In addition, patients with a pain domain of three or higher had a history of frequent shoulder joint dislocations.

There are several limitations to this research. First, the cross-sectional design of the study cannot determine causal relationships and clarify whether the clinical characteristics summarized by age are associated with growth. Second, we were unable to evaluate the laxity of joint capsules and ligaments qualitatively or quantitatively. Lastly, and most importantly, we were unable to systematically examine the shoulder joint and obtain their radiographs; thus, it is unclear to what extent the shoulder can potentially affect upper extremity function.

## 5. Conclusions

Dislocation of the elbow joint may have been induced by congenital laxity of the joint capsule or a shallow trochlear notch. In patients with elbow hyperextension, the use of braces that limit elbow extension may prevent elbow dislocation. In addition, due to the dislocation of the radial head and morphological abnormality of the proximal ulna, deformity of the elbow and wrist joints may progress and lead to further deterioration of upper extremity motor function. The findings of the present study also suggest an important role for dermatan sulfate in the maintenance of the skeletal system.

## Figures and Tables

**Table 1:** Demographic information.

Average Age (Years)	21 (4–45)
Sex	
Female	7
Male	6
History of fracture	
Yes	1
No	12
History of dislocation	
Yes	12
No	1
Dislocation site	
Shoulder	11
Elbow	2
Hip	0
Patella	7

**Table 2:** The QuickDASH questionnaire consists of eleven items with a score of 1 to 5 points for each item.

Question No.	Item
1	Open a tight or new jar
2	Do heavy household tasks (e.g., wash walls, wash floors)
3	Carry a shopping bag or briefcase
4	Wash your back
5	Use a knife to cut food
6	Recreational activities which require little effort (e.g., golf, hammering, tennis, etc.)
7	During the past week, to what extent has your arm, shoulder, or hand problem interfered with your normal social activities with family, friends, neighbors, or groups?
8	During the past week, were you limited in your work or other daily activities as a result of your arm, shoulder, or hand problem?
9	Arm, shoulder, or hand pain
10	Tingling (pins and needles) in your arm, shoulder, or hand
11	During the past week, how much difficulty have you had sleeping because of the pain in your arm, shoulder, or hand?

**Table 3:** Radiological features of patients with mcEDS.

Patient	Age	Sex	Elbow				Wrist		
			ACI	TDI	PUB	ROA	RD	VT	UV
1	4	F	1.36	-	19.4	-	-	-	-
2	6	M	1.58	-	2.5	-	-	-	-
3	11	M	2.01	-	18.4	-	20.2	13.9	−0.1
4	12	F	1.48	0.38	2.0	106	20.2	13.9	0.9
5	14	M	1.80	0.39	9.0	101	20.3	9.0	−1.9
6	16	F	1.40	0.32	10.0	113	27.0	15.5	0
7	18	M	1.96	0.40	0	104	27.7	18.3	−1.2
8	22	M	2.18	0.46	10.7	92	27.8	24.8	0
9	24	F	1.83	0.46	16.8	85	22.9	23.6	0.8
10	28	F	1.60	0.33	19.9	110	31.1	27.9	2.8
11	31	F	1.72	0.33	21.4	113	28.0	23.7	7.7
12	39	M	1.78	0.51	4.5	86	23.0	12.9	1.0
13	45	F	1.38	0.30	14.5	116	22.3	17.4	3.2

Anterior Coverage Index (ACI), Trochlear Depth Index (TDI), Proximal Ulnar Bow (PUB) and Radiographic Opening Angle (ROA) are derived from the elbow joint, and Radial deviation (RD), Volar tilt (VT) and Ulnar Variance (UV) were measured at the wrist.

**Table 4:** Mean length and width of the phalangeal and metacarpal bones in adults (mm).

			Males (n = 3)		Females (n = 4)	
			Length	Width	Length	Width
I	Metacarpal		47.3	7.8	43.5	7.4
	Phalanx	proximal	27.3	5.5	27.4	5.5
		distal	20.4	3.7	18.7	3.1
II	Metacarpal		64.4	7.7	59.8	6.8
	Phalanx	proximal	37.3	6.8	36.4	6.6
		middle	21.2	4.9	21.1	4.6
		distal	14.4	3.1	14.7	2.8
III	Metacarpal		63.0	6.6	56.1	6.1
	Phalanx	proximal	43.5	7.2	39.8	6.0
		middle	24.5	5.4	24.0	4.7
		distal	16.0	3.4	15.9	3.1
IV	Metacarpal		56.4	5.6	51.8	5.5
	Phalanx	proximal	40.4	6.0	37.7	5.4
		middle	23.7	4.7	23.3	4.4
		distal	16.6	3.0	16.0	3.3
V	Metacarpal		51.5	6.1	49.0	5.7
	Phalanx	proximal	31.4	5.3	29.9	4.8
		middle	16.3	3.7	16.4	3.6
		distal	13.8	2.2	13.1	2.3

**Table 5:** Mean width of the medullary cavity of the third metacarpal and ratio of medullary cavity of the metacarpal bone.

Year	Number of Patients	Width of Medullary Cavity (mm)	Ratio of Medullary Cavity of the Metacarpal Bone
Under 12	3	2.5	0.52
12	1	3.1	0.44
14	1	3.6	0.55
16	1	1.0	0.15
18	1	1.1	0.21
20–29	3	2.1	0.33
30–39	2	2.4	0.31
40–49	1	1.8	0.29

**Table 6:** QuickDASH score of patients with mcEDS.

Patient	QuickDASH Score	Component of QuickDASH										
		1	2	3	4	5	6	7	8	9	10	11
1	37.5	5	-	3	5	2	5	1	1	1	1	1
2	47.7	4	4	3	4	4	3	3	4	1	1	1
3	34.1	5	4	3	4	2	1	2	2	1	1	1
4	36.4	5	4	4	4	2	1	2	2	1	1	1
5	54.5	5	4	4	5	2	2	4	5	2	1	1
6	52.3	5	5	5	4	1	1	5	2	1	4	1
7	18.2	3	2	2	1	3	1	2	2	1	1	1
8	4.5	2	1	1	1	1	1	1	1	1	2	1
9	31.8	5	3	3	1	3	1	2	1	3	2	1
10	50	5	4	4	4	3	2	3	3	2	1	2
11	31.8	5	3	3	2	3	1	2	2	2	1	1
12	15.9	2	1	2	5	1	1	1	1	2	1	1
13	34.1	5	4	3	1	2	1	4	3	1	1	1

## Data Availability

The data presented in this study are available upon reasonable request from the corresponding author. The data are not publicly available due to privacy restrictions.
